# Supplementary material for: Complexome profiling of the Chlamydomonas psb28 mutant reveals TEF5 as an early PSII assembly factor
Source: Plant Cell. 2025 Mar 17;37(6):koaf055. doi: 10.1093/plcell/koaf055 (PMC12164590; doi:10.1093/plcell/koaf055)
Supplement: koaf055_Supplementary_Data [file koaf055_supplementary_data.zip › psb28_interactive.zip!/plots/intensity/qualityPlots/peptides/SGA1_Cre01.g005150.t1.1.html]

### Cre01.g005150.t1.1

Trivial name: SGA1  
SeqCoverage [median]: 22.50  
Mass: 48.79 kDa  
  
 

| Sequence | MissedCleavage | Mass [Da] | wtIntensity | muIntensity | Score |
| --- | --- | --- | --- | --- | --- |
| AMHVPGQNHR | 0 | 1145.55 | 0.00 | 774.82 | 92.05 |
| AMSLPTGLAFVAASPK | 0 | 1559.84 | 22451.66 | 20791.13 | 156.69 |
| ATEYWHK | 0 | 933.43 | 0.00 | 1055.11 | 151.17 |
| NHLFVPGPVNIHER | 0 | 1627.86 | 9913.88 | 26070.37 | 170.95 |
| SDSLTVVEVPEGVDSNK | 0 | 1773.86 | 9430.10 | 12391.54 | 210.79 |
| SDSLTVVEVPEGVDSNKIVK | 1 | 2114.11 | 4669.64 | 12815.41 | 99.41 |
| VDVAVTGSQK | 0 | 1002.53 | 13779.24 | 12414.09 | 163.16 |
| VYYDFADMLR | 0 | 1291.59 | 16754.72 | 28167.83 | 138.40 |
| WRSDSLTVVEVPEGVDSNK | 1 | 2116.04 | 459.82 | 1755.60 | 64.51 |
|  |  |  | 77459.1 | 116235.9 |  |
